# The Health and Welfare of Rural and Urban Cancer Survivors in Missouri

**DOI:** 10.5888/pcd10.130052

**Published:** 2013-09-12

**Authors:** Mario Schootman, Sherri Homan, Kathryn E. Weaver, Donna B. Jeffe, Shumei Yun

**Affiliations:** Author Affiliations: Sherri Homan, Shumei Yun, Missouri Department of Health and Senior Services, Office of Epidemiology, Jefferson City, Missouri; Kathryn E. Weaver, Wake Forest School of Medicine, Winston-Salem, North Carolina; Donna B. Jeffe, Washington University School of Medicine, Saint Louis, Missouri.

## Abstract

**Introduction:**

An estimated 2.8 million cancer survivors reside in rural areas in the United States. We compared the risk behaviors, psychosocial factors, health outcomes, quality of life, and follow-up care of rural and urban cancer survivors in Missouri.

**Methods:**

We used 2009–2010 Missouri Behavioral Risk Factor Surveillance System data to examine various health outcomes, behaviors, and psychosocial factors among rural and urban cancer survivors and their respective rural and urban counterparts without a cancer history. Cancer survivors also were asked about receipt of survivorship care plan components. Sociodemographic factors, access to medical care, and chronic conditions were examined as potential explanatory factors for differences among the 4 groups.

**Results:**

An estimated 9.4% of rural and 7.9% of urban Missourians aged 18 years or older reported a cancer history. Rural survivors reported the highest rates of poor self-reported health, physical distress, and activity limitation; however differences between rural and urban survivors were attributable largely to sociodemographic differences. Both rural and urban cancer survivors reported more fatigue than their respective counterparts without a cancer history. Rural survivors also were less likely to meet Centers for Disease Control and Prevention recommendations for physical activity than their rural controls. The prevalence of smoking among rural survivors was higher than among urban survivors. Only 62% of rural survivors versus 78% of urban survivors reported receiving advice about cancer follow-up care.

**Conclusion:**

Rural cancer survivors face many health challenges. Interventions to improve quality of life and health behaviors should be adapted to meet the needs of rural cancer survivors.

## Introduction

An estimated 13.7 million cancer survivors in the United States are at risk of recurrence, second primary cancers, and late effects of treatment, all of which can adversely affect their health status, physical and psychosocial functioning, quality of life, and survival (1). An estimated 2.8 million cancer survivors in the United States reside in rural areas and may be affected by geographic barriers associated with rural residence (2). Compared with people who live in urban areas, people who live in rural areas may have more financial burdens, labor-intensive work, social isolation, stress, and lower accessibility to health care, which may affect health behaviors and health outcomes (3,4). These challenges may be greater among rural residents with a history of poor health or chronic conditions, such as cancer, who face longer travel times and lower access to specialized care.

Although a growing literature focuses on the medical follow-up of cancer survivors (5,6), little attention has focused on the systematic assessment of behavioral risk factors that are independent predictors of recurrence, second primary cancers, reduced survival, and poorer quality of life (7–9), which may contribute to rural–urban differences in outcomes after cancer. Survivorship care plans may play a key role in improving cancer survivors’ health behaviors, quality of life, and survival (10). In 2006, the Institute of Medicine recommended that patients should be provided a comprehensive care summary and follow-up plan (ie, Survivorship Care Plan) that summarizes their cancer diagnosis, types of treatment received, timing of and expectations for recommended follow-up, and recommendations for engagement in health-promoting behaviors (11). The Commission on Cancer mandates that all organizations must create a formal plan for the delivery of survivorship care plans by 2014 and implement that plan by 2015 to maintain accreditation (12). Little is known about rural–urban differences in the use of survivorship care plans.

We determined the prevalence of unhealthful lifestyle behaviors, poor psychosocial factors, and adverse health outcomes among Missouri’s rural and urban cancer survivors by conducting a cross-sectional analysis of data from the 2009–2010 Behavioral Risk Factor Surveillance System (BRFSS). Because of the potential inherent differences between rural and urban populations, we also compared rural and urban survivors with their respective rural and urban populations without a history of cancer. Regional variation in health behaviors, psychosocial factors, and outcomes may be obscured by national-level analyses. Also, because many public health and policy interventions are implemented at the state level, detailing survivors’ behaviors in 1 state may better direct finite resources.

## Methods

### Data source and questions

The BRFSS is a continuous telephone interview covering a variety of health-related topics, providing estimates of the civilian noninstitutionalized population. For the 2009 Missouri BRFSS, the Council of American Survey Research Organizations (CASRO) response rate was 57.4%. For the 2010 Missouri BRFSS, the CASRO response rate was 59.5% (13). Most questions were asked in both 2009 and 2010, but some were asked only in 2009 or 2010.

Participants were asked “Have you ever been told by a doctor or other health professional that you had cancer?” Follow-up questions included number of cancers and cancer type(s). Cancer survivors were compared with people who reported no cancer history (controls). Similar to the methods of other studies, we excluded participants who reported nonmelanoma skin cancer and who did not complete the cancer survivorship questions (14,15). The county of residence at the time of the interview was coded according to its location in a metropolitan statistical area (MSA) as defined by the US Office of Management and Budget. Counties were coded as urban if they were located in an MSA; otherwise, they were coded as rural.

Self-rated health was measured using the question, “How would you rate your health — would you say it is excellent, very good, good, fair, or poor?” Self-rated health was dichotomized into “fair or poor” versus “excellent, very good, or good.” Frequency of physical and mental distress was assessed by asking participants to report separately the number of days in the past month when their physical and mental health was “not good,” which was dichotomized into less than 14 days versus at least 14 days. Activity limitations were measured by asking participants to report the number of days in the past month when their poor physical or mental health kept them from doing their usual activities, which was dichotomized into less than 14 days versus at least 14 days. Participants rated their life satisfaction on a scale from very satisfied to very dissatisfied, which was used as a continuous variable. In 2010, a single question assessed the prevalence and frequency of fatigue, asking about how many days the participant felt tired or had little energy in the last 14 days, which was dichotomized into less than 7 days per 2 weeks versus at least 7 days per 2 weeks.

Respondents were asked about their physical activity with the question, “During the past month . . . did you participate in any physical activities such as running, calisthenics, golf, gardening, or walking for exercise?” During 2009, participants were asked more details about their physical activity, and we compared participants who met Centers for Disease Control and Prevention (CDC) physical activity recommendations with those who did not. Smoking status was classified as being a current smoker or a former or never smoker. To measure overuse of alcohol consistent with public health guidelines and previous research, we categorized alcohol intake as 0 to 1 drink per day versus more than 1 drink per day for women and 0 to 2 drinks per day versus more than 2 drinks per day for men (16). We compared participants who ate at least 5 servings of fruits or vegetables per day with those who did not. To assess the number of hours slept per night, the BRFSS asked, “On average, how many hours of sleep do you get in a 24-hour period?” Body mass index (BMI) was calculated based on self-reported height and weight. In 2009, participants also reported on aspirin use every day or every other day (17). In 2010, the Patient Health Questionnaire (PHQ-8) was used to assess the prevalence and severity of depression (18). In 2010, perceived emotional support was obtained from the question “How often do you get the social and emotional support you need?”

During 2010, participants were asked about aspects of a Survivorship Care Plan, including receiving 1) a written summary of the cancer treatments they received, and 2) advice about where they should go or where they should receive routine follow-up cancer checkups from a doctor, nurse, or other health professional. Participants also were asked whether their health insurance paid for all or part of their cancer treatment, denial of health or life insurance because of their cancer history, participation in a clinical trial as part of their cancer treatment, and current physical pain caused by cancer or its treatment.

On the basis of previous research, we selected the following factors to include as covariates in our analysis: 1) sociodemographic factors (age, race/ethnicity, sex, annual household income categories, employment status, marital status), 2) access to medical care (having health insurance, having a checkup within the past year, unable to see a doctor because of cost), and 3) number of chronic conditions (ie, diabetes, heart disease, stroke, asthma) (19–21). We used county-level data from the Area Resource File (number of surgeons per population, number of primary care physicians per population), Bureau of the Census (poverty rate), Department of Agriculture (number of fitness or recreation centers per population, percentage of people with low income and who live more than 1 mile from a supermarket or large grocery store if in an urban area or more than 10 miles from a supermarket or large grocery store if in a rural area), and average monthly dollar amount of the Supplemental Nutrition Assistance Program benefits per population.

### Statistical analysis

We calculated the prevalence of the health status and behavioral risk factor measures by cancer history (cancer survivors vs noncancer controls) and rural–urban location. Model 1 is unadjusted; Model 2 adjusted for sociodemographic characteristics; Model 3 adjusted for sociodemographic characteristics and access to medical care; Model 4 adjusted for sociodemographic characteristics and chronic conditions; and Model 5 adjusted for sociodemographic characteristics and county-level data. We used multivariable logistic regression to compute adjusted proportions of the dependent variables, called predicted marginals, for each of the 4 groups; the predicted marginals can be interpreted as conditional prevalence estimates. An advantage of using predicted marginals over traditional odds ratios is that the former approach does not require the use of a reference group against which all other groups are compared. Pairwise least squares mean differences were also calculated and their significance tested using the z test. Variance estimates were calculated using the Taylor series approximation. The covariates were included as groups of variables in the logistic regression models to examine their effects on the predicted marginals. To obtain representative estimates, all data were weighted by adjusting for the probability of inclusion in the sample. We used SAS version 9.3 (SAS Institute, Inc, Cary, North Carolina) for data analysis.

## Results

The 2009–2010 Missouri BRFSS data set included 9,530 adults (4,497 in 2009 and 5,033 in 2010) after excluding 956 participants because they did not complete the cancer survivor module, reported they did not know or refused to answer the question about having ever had cancer, or reported having nonmelanoma skin cancer. An estimated 8.3% of participants reported a lifetime history of cancer (urban: 7.9%; rural: 9.4%). Of all cancer survivors, 29.9% lived in rural counties.

The proportion of female gynecological cancers and melanoma was significantly higher among rural residents (Figure), and the proportion of breast, colorectal, and prostate cancers was higher among urban residents (*P* = .01). More urban survivors (12.7%) compared with rural survivors (9.0%) were still receiving cancer treatment (surgery, radiation therapy, chemotherapy, or chemotherapy pills) (*P* = .03). There was no difference between rural and urban residents in number of lifetime cancer diagnoses or average time since diagnosis. The age distribution among survivors was similar across participant location (Table 1). A greater proportion of rural (57.5%) than urban (46.1%) survivors had at least 1 comorbid condition.

**Figure:**
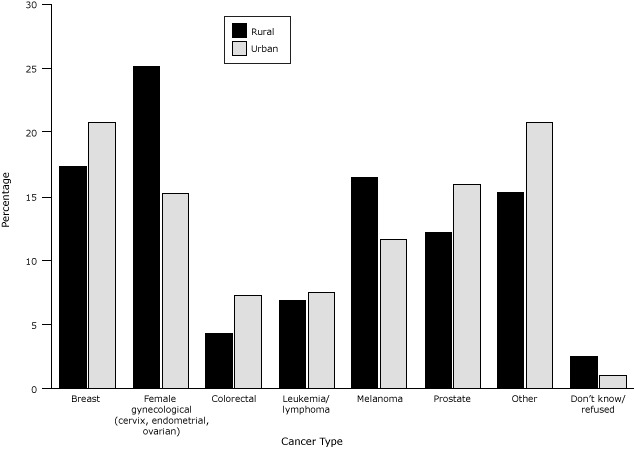
Proportion of cancers among urban and rural cancer survivors, by cancer type, Missouri Behavioral Risk Factor Surveillance System, 2009–2010. Other cancer includes bladder, head/neck, esophageal, liver, pancreatic, stomach, testicular, lung, renal, bone, brain, neuroblastoma, and all other cancers. **Table:** 

Cancer Type	Urban	Rural
Breast	20.8	17.3
Female gynecological (cervix, endometrial, ovarian)	15.2	25.1
Colorectal	7.2	4.3
Leukemia/lymphoma	7.5	6.9
Melanoma	11.7	16.5
Prostate	15.9	12.2
Other	20.8	15.3
Don’t know/refused	1	2.5

**Table 1:** Sociodemographic Characteristics of Study Population, Study of Health and Welfare of Cancer Survivors, by Rural–Urban Location, Missouri 2009–2010

Characteristic	Rural		Urban	
	Survivor (n = 471)	Control (n = 3,088)	Survivor (n = 701)	Control (n = 5,270)
Weighted n	101,923	977,825	239,496	2,793,265
**Sociodemographics**				
**Age, y**				
18–44	17.9	49.5	10.2	51.3
45–64	33.0	32.2	41.5	34.1
65–74	22.5	10.2	21.4	8.1
≥75	25.7	7.8	26.4	6.1
Unknown	0.9	0.3	0.5	0.4
**Sex**				
Male	36.3	49.2	40.4	48.5
Female	63.7	50.8	59.6	51.5
**Race/ethnicity**				
Non-Hispanic white	90.4	91.5	82.5	81.1
Non-Hispanic black	0.9	1.8	8.9	11.0
Hispanic	1.6	2.1	0.6	3.2
Other non-Hispanic	1.0	2.6	4.9	2.3
Multiracial	6.0	2.0	3.1	2.4
**Household income, $**				
<15,000	14.2	10.8	6.5	6.7
15,000–24,999	19.1	17.4	16.5	12.0
25,000–34,999	14.9	10.8	11.8	10.1
35,000–49,999	15.6	17.8	12.9	14.6
≥50,000	21.4	30.1	35.0	44.1
Unknown	15.0	13.1	17.2	12.6
**Health care access**				
**Unable to see doctor because of medical cost**				
Yes	17.2	15.4	10.6	13.0
No	82.8	84.5	89.1	86.9
Unknown	0.0	0.1	0.4	0.1
**Number of chronic conditions**				
0	42.5	73.5	53.9	75.7
1	38.9	19.8	28.3	19.4
≥2	18.5	6.7	17.8	4.9

### Health status

The prevalence of fair or poor health was 38.5% among rural survivors compared with 27.4% among urban survivors and less than 20% among both control groups (Model 1, each *P* < .05) (Table 2). After controlling for sociodemographic characteristics (Model 2), prevalence of fair or poor health was similar for rural and urban survivors but was higher compared with rural and urban controls. Adding access to medical care (Model 3) did not affect the differences among the 4 groups. Adding the number of chronic conditions (Model 4) to sociodemographic characteristics (Model 2) reduced differences between rural survivors and rural controls, suggesting that differences in the number of chronic conditions explained the initial differences between both groups. There was no significant difference in frequent physical distress between rural and urban survivors (Model 1), but prevalence was higher when comparing rural survivors (29.1%) with rural controls (13.2%) and when comparing urban survivors (23.5%) with urban controls (9.1%). Only the difference between urban survivors and controls remained significant in fully adjusted models. The prevalence of frequent activity limitations was higher among rural survivors (22.8%) than among urban survivors (15.7%) and among rural controls (8.4%). Prevalence of frequent activity limitations also was higher when comparing urban survivors (15.7%) with urban controls (6.8%). After controlling for sociodemographic characteristics, differences remained when comparing rural and urban survivors with rural and urban controls, respectively. Access to medical care and number of chronic conditions did not further explain any differences. Rural survivors (48.8%) had a higher prevalence of fatigue than rural controls (27.9%), and urban survivors (36.1%) reported more fatigue than urban controls (26.5%). These differences persisted in adjusted models. Overall, life satisfaction was 1.6% on a 4-point scale, with no differences across the 4 groups. Adding the county-level variables (Model 5) to the model with sociodemographic characteristics (Model 2) did not change any of the results.

**Table 2:** Self-Rated Health of Cancer Survivors and Noncancer Controls, by Rural or Urban Location, Behavioral Risk Factor Surveillance System, Missouri, 2009–2010

Location	Model 1^a^^,^ b	Model 2^a^	Model 3^a^	Model 4^a^	Model 5^a^

	% (Standard Error)				
**Fair or poor self-rated health**					
Rural, survivor	38.5 (2.8)^c^^,^ d ^,^ e	29.5 (3.0)^d^^,^ e	27.3 (3.0)^d^^,^ e	24.2 (3.0)^e^	27.3 (3.1)^d^^,^ e
Rural, control	19.6 (1.0)	20.9 (1.2)	19.8 (1.2)	20.4 (1.2)	24.9 (2.5)
Urban, survivor	27.4 (2.1)	23.5 (2.4)	22.2 (2.4)	20.0 (2.3)	19.2 (1.4)
Urban, control	13.0 (0.7)	14.3 (0.8)	13.6 (0.7)	13.9 (0.8)	15.1 (0.8)
**Frequent physical distress (≥14 days in past month)**					
Rural, survivor	29.1 (2.9)^d^^,^ e	19.1 (2.4)^d^^,^ e	17.9 (2.3)^d^^,^ e	15.6 (2.2)^e^	17.0 (2.4)^d^^,^ e
Rural, control	13.2 (0.8)	13.1 (0.9)	12.7 (0.9)	12.6 (0.9)	16.8 (2.1)
Urban, survivor	23.5 (2.3)	15.7 (2.0)	15.0 (2.0)	13.2 (1.8)	11.9 (1.1)
Urban, control	9.1 (0.5)	9.6 (0.7)	9.3 (0.7)	9.2 (0.7)	10.1 (0.7)
**Frequent mental distress (≥14 days in past month)**					
Rural, survivor	15.0 (2.7)	11.5 (2.3)	10.4 (2.0)	10.1 (2.1)	10.6 (2.2)
Rural, control	12.4 (0.9)	10.5 (8.2)	9.9 (0.8)	10.4 (0.8)	12.0 (1.9)
Urban, survivor	13.0 (2.0)	11.7 (1.9)	11.0 (1.9)	10.7 (1.7)	9.6 (1.0)
Urban, control	12.2 (0.8)	10.6 (0.7)	10.2 (0.6)	10.6 (0.7)	10.9 (0.7)
**Frequent activity limitation (≥14 days in past month)**					
Rural, survivor	22.8 (3.0)^c^^,^ d ^,^ e	11.2 (2.1)^d^^,^ e	10.1 (1.9)^d^^,^ e	9.5 (1.8)^d^^,^ e	9.8 (2.0)^d^^,^ e
Rural, control	8.4 (0.7)	5.7 (0.6)	5.2 (0.5)	5.5 (0.6)	8.9 (1.5)
Urban, survivor	15.7 (2.1)	8.3 (1.4)	7.7 (1.4)	7.3 (1.3)	5.0 (0.7)
Urban, control	6.8 (0.5)	5.1 (0.5)	4.8 (0.5)	5.0 (0.4)	5.5 (0.5)
**Fatigue (≥7 days/2 weeks)^f^**					
Rural, survivor	48.8 (6.0)^d^^,^ e	43.8 (5.5)^d^^,^ e	41.6 (5.4)^d^^,^ e	40.1 (5.4)^d^^,^ e	39.3 (5.7)^d^^,^ e
Rural, control	27.9 (1.8)	26.3 (1.9)	25.4 (1.9)	26.3 (1.9)	38.8 (4.3)
Urban, survivor	36.1 (4.2)	37.8 (4.2)	37.9 (4.3)	36.0 (4.3)	24.2 (2.3)
Urban, control	26.5 (1.5)	24.9 (1.2)	24.7 (1.2)	24.9 (2.1)	25.3 (1.4)

a Model 1 is unadjusted; Model 2 adjusted for sociodemographic characteristics (age group, Hispanic origin, sex, household income categories, employment, and marital status); Model 3 adjusted for sociodemographic characteristics and access to medical care (ie, having health care insurance at the time of the interview, being unable to see a doctor during the 12 months before the interview because of cost, and having a routine checkup within 1 year); Model 4 adjusted for sociodemographic characteristics and chronic conditions (chronic conditions covariate was the sum of conditions based on participants’ self-reported diagnosis by a physician, including diabetes, heart attack, coronary heart disease, stroke, asthma, and more than 1 cancer); and Model 5 adjusted for sociodemographic characteristics and county-level data. Overall percentage is shown on the first line for each variable.

b Percentages and standard errors overall for each category are as follows: fair–poor self-rated health, 16.3 (0.6); 14 or more days of frequent physical distress in the past month, 11.3 (0.5); 14 or more days of frequent mental distress in the past month, 12.2 (0.6); 14 or more days of frequent activity limitation in the past month, 8.1 (0.4); and 7 or more days of fatigue for 2 weeks, 27.8 (1.2).

c *P* < .05 for comparison between rural and urban survivors.

d *P* < .05 for comparison between rural survivors and rural controls.

e *P* < .05 for comparison between urban survivors and urban controls.

f Only asked in 2010.

### Health behaviors

Although there was no significant difference in physical activity between rural (60.3%) and urban (64.0%) survivors, both groups were less likely to report physical activity compared with their no-cancer controls (Table 3). After controlling for covariates, the only difference in physical activity prevalence that remained significant was between urban survivors and urban controls. Although there were no significant differences between rural and urban survivors in meeting the CDC physical activity recommendation in 2009, rural survivors were less likely to meet these recommendations than were rural controls. This was the only significant difference among the 4 subgroups even when controlling for covariates. 

**Table 3:** Body Mass Index and Health Behaviors of Cancer Survivors and Non-Cancer Controls, by Location, Missouri 2009–2010

Location	Model 1^a^^,^ b	Model 2^a^	Model 3^a^	Model 4^a^	Model 5^a^

	% (Standard Error)				
**Any physical activity**					
Rural, survivor	60.3 (3.2)^c^^,^ d	63.1 (3.1)^d^	63.8 (3.1)^d^	64.6 (3.1)^d^	63.6 (3.3)^d^
Rural, control	69.2 (1.2)	65.4 (1.3)	66.2 (1.3)	65.3 (1.3)	64.7 (2.8)
Urban, survivor	64.0 (2.7)	65.6 (2.7)	65.3 (2.7)	66.5 (2.7)	65.6 (1.7)
Urban, control	76.1 (0.9)	72.3 (0.9)	72.4 (0.9)	72.2 (0.9)	71.5 (1.0)
**Physical activity (meeting CDC recommendation)** e					
Rural, survivor	34.5 (4.4)^c^^,^ d	36.9 (4.4)^c^	36.9 (4.4)^c^	37.7 (4.4)^c^	37.1 (4.6)^c^
Rural, control	52.8 (1.9)	48.0 (1.9)	48.1 (1.9)	47.9 (1.9)	43.0 (3.9)
Urban, survivor	40.8 (3.7)	42.8 (3.9)	42.8 (3.9)	43.4 (3.9)	47.6 (2.3)
Urban, control	50.3 (1.5)	44.9 (1.4)	44.9 (1.4)	44.6 (1.4)	43.9 (1.6)
**Current smoker**					
Rural, survivor	24.9 (3.1)^d^^,^ f	26.1 (3.3)^f^	24.7 (3.3)^f^	25.7 (3.3)^f^	23.2 (3.2)
Rural, control	25.1 (1.2)	22.0 (1.2)	20.7 (1.1)	22.0 (1.2)	17.2 (2.1)
Urban, survivor	14.8 (1.6)	16.0 (2.0)	15.7 (2.0)	15.8 (2.0)	19.5 (1.4)
Urban, control	20.8 (0.9)	18.5 (0.8)	18.1 (0.8)	18.5 (0.8)	19.5 (0.9)
**Excessive alcohol use**					
Rural, survivor	2.2 (0.9)	2.3 (0.9)	2.4 (0.9)	2.4 (1.0)	2.4 (1.1)
Rural, control	4.8 (0.6)	3.6 (0.5)	3.5 (0.5)	3.6 (0.5)	3.1 (0.9)
Urban, survivor	3.6 (0.9)	3.8 (1.0)	3.9 (1.0)	3.9 (1.0)	4.0 (0.7)
Urban, control	5.0 (0.5)	3.8 (0.4)	3.8 (0.4)	3.8 (0.4)	3.3 (0.4)
**Fruit and vegetables ≥5/d^e^**					
Rural, survivor	21.6 (3.1)	19.3 (3.0)	18.7 (3.0)	19.1 (3.0)	21.1 (3.4)
Rural, control	18.7 (1.4)	20.6 (1.5)	20.7 (1.5)	20.6 (1.5)	20.3 (3.1)
Urban, survivor	24.8 (3.3)	21.7 (3.2)	21.3 (3.2)	21.6 (3.3)	22.6 (1.9)
Urban, control	19.8 (1.2)	21.8 (1.2)	21.5 (1.1)	21.8 (1.2)	19.9 (1.3)
**Sleeping <7 h/night^e^**					
Rural, survivor	39.0 (6.3)	37.3 (5.7)	36.0 (5.7)	35.2 (5.6)	35.5 (5.8)
Rural, control	33.1 (1.9)	30.3 (1.8)	29.8 (1.8)	30.4 (1.8)	38.3 (4.3)
Urban, survivor	35.7 (4.0)	37.8 (4.2)	38.0 (4.2)	36.8 (4.2)	28.1 92.3)
Urban, control	34.3 (1.5)	31.2 (1.3)	31.0 (2.3)	31.2 (1.3)	31.7 (1.5)
**Body mass index ≥25 kg/m^2^**					
Rural, survivor	65.9 (3.6)	67.1 (3.3)	66.9 (3.2)	65.3 (3.5)	66.4 (3.5)
Rural, control	65.9 (1.3)	69.5 (1.3)	70.0 (1.3)	69.9 (1.3)	66.8 (2.7)
Urban, survivor	65.3 (2.6)	65.4 (2.7)	64.8 (2.8)	64.5 (2.7)	68.8 (1.6)
Urban, control	62.9 (1.1)	67.7 (0.9)	67.7 (1.0)	68.3 (1.0)	68.8 (1.1)
**Aspirin use every (other) day**					
Rural, survivor	55.4 (4.8)^c^^,^ d	39.6 (5.6)^d^	37.9 (5.4)	35.8 (5.3)	37.5 (5.8)
Rural, control	28.3 (1.5)	35.9 (2.1)	35.9 (2.1)	36.2 (2.1)	42.3 (4.6)
Urban, survivor	53.9 (4.1)	41.1 (4.5)	40.1 (4.5)	39.4 (4.9)	34.2 (2.5)
Urban, control	24.0 (1.2)	32.2 (1.6)	31.8 (1.6)	33.0 (1.6)	34.5 (1.9)

Abbreviation: CDC, Centers for Disease Control and Prevention.

a Model 1 is unadjusted; Model 2 adjusted for sociodemographic characteristics (age group, Hispanic origin, sex, household income categories, employment, and marital status); Model 3adjusted for sociodemographic characteristics and access to medical care (ie, having health care insurance at the time of the interview, being unable to see a doctor during the 12 months before the interview because of cost, and having a routine checkup within 1 year); Model 4 adjusted for sociodemographic characteristics and chronic conditions (chronic conditions covariate was the sum of conditions based on participants’ self-reported diagnosis by a physician, including diabetes, heart attack, coronary heart disease, stroke, asthma, and more than 1 cancer); and Model 5 adjusted for sociodemographic characteristics and county-level data. Overall percentage is shown on the first line for each variable.

b Percentages and standard errors overall for each category as follows: any physical activity, 73.3 (0.7); physical activity meeting CDC recommendation, 49.9 (1.2); current smoker, 21.6 (0.7); excessive alcohol use, 5.0 (0.4); fruit and vegetable intake ≥5 servings per day, 19.9 (0.9); sleeping <7 h per night, 34.2 (1.2); body mass index ≥25.0 kg/m^2^, 63.8 (0.8); and aspirin use every (other) day, 25.0 (0.9).

c *P* < .05 for comparison between rural survivors and rural controls.

d *P* < .05 for comparison between urban survivors and urban controls.

e Only asked in 2009.

f *P* < .05 for comparison between rural and urban survivors.

Rural survivors (24.9%) were more likely to smoke than urban survivors (14.8%) but equally likely compared with rural controls. Urban survivors (14.8%) were less likely to smoke compared with urban controls (20.8%), but this difference was due to differences in sociodemographic characteristics (Model 2). Access to medical care and chronic conditions did not further alter the findings. There were no significant differences among the 4 groups for excessive alcohol use, fruit and vegetable consumption, hours of sleep, or overweight or obesity. Although there were no significant differences in aspirin use between rural and urban survivors, both survivor groups were more likely to take aspirin compared with rural and urban controls, respectively. Controlling for access to medical care or chronic conditions made all differences in aspirin use among the groups disappear. Adding the county-level variables (Model 5) to the model with sociodemographic characteristics (Model 2) did not change any of the results.

### Psychosocial factors

Although rural survivors had higher levels of depression (20.9%) than both urban survivors (10.5%) and rural controls (10.5%), there were no differences among the 4 groups when controlling for sociodemographic characteristics, access to medical care, or chronic conditions. The percentage who reported getting social and emotional support when needed did not vary significantly across the 4 subgroups.

### Cancer survivorship issues

Of cancer survivors, 26.4% reported receiving a written treatment summary; no significant difference was found between urban (28.9%) and rural (20.6%) participants (*P* = .48). Rural survivors (61.7%) were less likely to receive follow-up care instructions than urban (78.2%) survivors (*P* = .02). There were no significant differences in lack of insurance for cancer treatment between urban and rural survivors, denial of health or life insurance coverage because of cancer, clinical trial participation, or cancer-related physical pain.

## Discussion

Although the number of cancer survivors is increasing rapidly (22), disparities in health status, behaviors and use of a survivorship care plan as a function of residential location have been understudied. Much of the research on health and behavioral outcomes among cancer survivors has focused on urban populations that have been treated at single institutions or on national studies that mask regional variations. In Missouri alone, an estimated 29.9% of all survivors lived in rural counties, a sizeable population. Rural cancer survivors in Missouri face considerable challenges after cancer, including less common types of cancer for which services may not be available locally, a larger number of comorbid conditions, lower household income, and lower access to medical care, compared with their urban counterparts. These challenges may explain some of the differences in health and behavioral outcomes between rural and urban survivors, but they also can create obstacles for the design and implementation of interventions aimed at improving these outcomes.

Of the 4 groups, rural survivors reported the highest rates of poor self-reported health, frequent physical distress, frequent activity limitation, fatigue, and depression; however differences between rural and urban survivors appear to be largely attributable to sociodemographic differences. This finding contrasts with those of other studies that observed persistent differences in health status among rural and urban survivors in a national sample (2). Our Missouri findings are consistent with US population-based studies that have observed higher rates of disability, higher rates of activity limitations, and poorer health status among cancer survivors than among adults without cancer (21,23).

Several differences and similarities existed in prevalence of behaviors between rural survivors and their rural counterparts without a cancer history. It is concerning that a large proportion of survivors, particularly those in rural counties, were not meeting CDC recommendations for physical activity, given the substantial benefits that adequate physical activity confers after cancer (24). Although rural populations face unique challenges, in general, to participate in physical activity (25), rural survivors may have even more barriers. Home-based exercise programs have shown promise among cancer survivors and may be more accessible to rural survivors (26). Particularly worrisome is the high prevalence of current smokers among rural survivors. Although evidence-based treatment options are available for smoking cessation, more innovative approaches may be needed to increase quit rates in rural areas, where treatment options may be limited (27). According to the 2011 Missouri County-Level Study, a higher percentage of smokers living in urban areas (49.8%) than in small towns or isolated rural areas (44.0%) had health insurance that covers cessation counseling or medication.

New models of care may need to be developed to maximize health outcomes for rural survivors (28). Coordination between oncologists and primary care providers may be challenging for rural survivors, who may receive their cancer treatment in an urban location far from home and then return to their local primary care provider for follow-up care (11). Survivorship care plans may help facilitate this transition. Only 26.4% of Missouri cancer survivors stated they had received a summary of the treatments they received, which is substantially lower than the 40.2% reported from a recent representative sample of 10 states and underscores the wide variation in the United States (29,30). Fewer rural survivors were told where to receive routine follow-up or whom to see after their cancer diagnosis, although our overall rate of follow-up of 73.0% is similar to recent national estimates (29). Nearly 1 of every 9 cancer survivors in Missouri reported having been denied health or life insurance because of their cancer history, which is similar to recent estimates from 10 states (29). Having been denied insurance may have additional impact on the types of medical services received by survivors, including follow-up for their previous cancer.

Limitations of the study include the BRFSS restriction to noninstitutionalized Missouri adults; self-reported cancer history; lack of information about types of treatment received, stage at diagnosis, and length of residence. Our findings show the unique situation of rural cancer survivors in terms of health outcomes, behavior, and cancer follow-up care, and suggest the need for interventions aimed at reducing disparities in cancer outcomes among the growing population of survivors.
